# Supplementary material for: A global screening identifies chromatin-enriched RNA-binding proteins and the transcriptional regulatory activity of QKI5 during monocytic differentiation
Source: Genome Biol. 2021 Oct 14;22:290. doi: 10.1186/s13059-021-02508-7 (PMC8518180; doi:10.1186/s13059-021-02508-7)

Additional file 1: Supplementary Figures

Figure S1

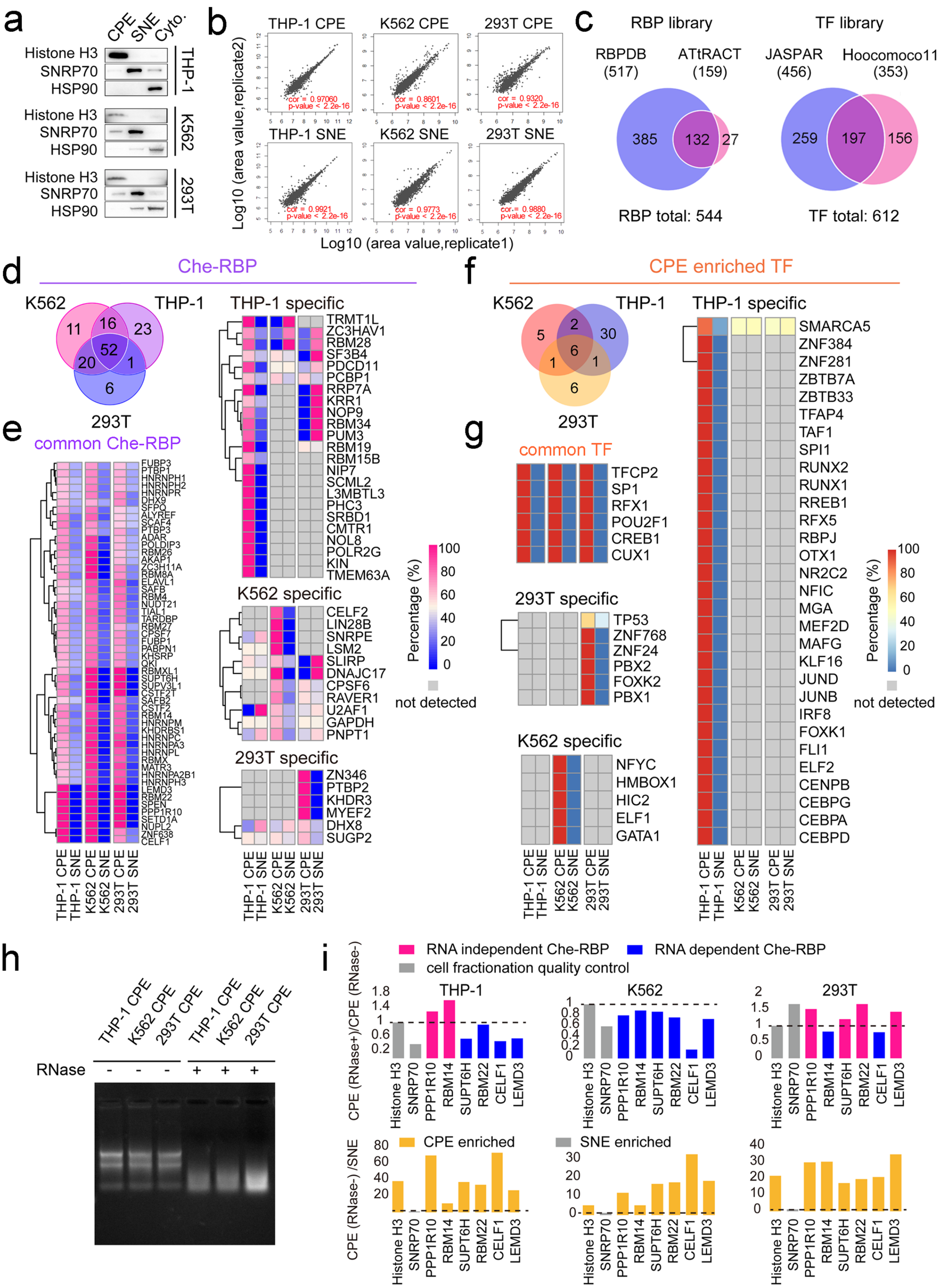

Figure S1. MS data analysis of nuclear RBPs in THP-1, K562 and 293T cells. a Validation of sub-nuclear fractionation Western blot, where Histone H3, SNRP70 and HSP90 act as positive controls for CPE fraction, SNE fraction and cytoplasm fraction, respectively. **b** Repeatability test of MS results. Pearson correlation coefficient was used to evaluate the repeatability of two biology replicates in CPE and SNE MS data. **c** Construction of the RBP (left) and TF (right) libraries from their respective databases. **d** Venn diagram showing the overlap of chromatin-enriched RBPs among K562, THP-1 and 293T cells. **e** Heatmap showing the shared (left panel) and specific (specifically expressed and/or specifically distributed) Che-RBPs (right panel) among the 3 cell lines. The color gradient indicates the percentage of protein abundance in CPE or SNE fractions of each cell line. Grey represents not detected. **f** Venn diagram showing the overlap of TFs expressed among the 3 cell lines. **g** Heatmap showing the shared and specific (specifically expressed or/and specifically distributing) TFs among the 3 cell lines. The color gradient indicates the percentage of protein abundance in CPE or SNE fractions of each cell line. Grey represents not detected. **h** Efficiency test for RNase treatment in sub-nuclear fractionation assay. CPE fractions from 3 cell lines were extracted and subjected to RNase A treatment for 5min at 4°C, when RNase-free samples were treated in the same condition without RNase. The agarose gel picture shows the efficiency of digestion. **i** Comparison of protein level quantified by analysis of immuno-blot results in Figure 1h. RNA-dependency of Che-RBPs' deposition on chromatin was calculated as the ratio of $\frac{\text{Protain CPE (RNase+)/Protein CPE (RNase−)}}{\text{Histone H3 CPE (RNase+)/Histone H3 CPE(RNase−)}}$. RNA-independent Che-RBPs are indicated by pink bars with $\frac{\text{Protain CPE (RNase+)/Protein CPE (RNase−)}}{\text{Histone H3 CPE (RNase+)/Histone H3 CPE(RNase−)}}>1$. RNA-dependent Che-RBPs are indicated by blue bars with $\frac{\text{Protain CPE (RNase+)/Protein CPE (RNase−)}}{\text{Histone H3 CPE (RNase+)/Histone H3 CPE(RNase−)}}<1$ (upper panel). Sub-nuclear localization of Che-RBPs was determined by $\frac{\text{Protein CPE (RNase−)}}{\text{Protein SNE}} > 1$ (lower panel).

Figure S2

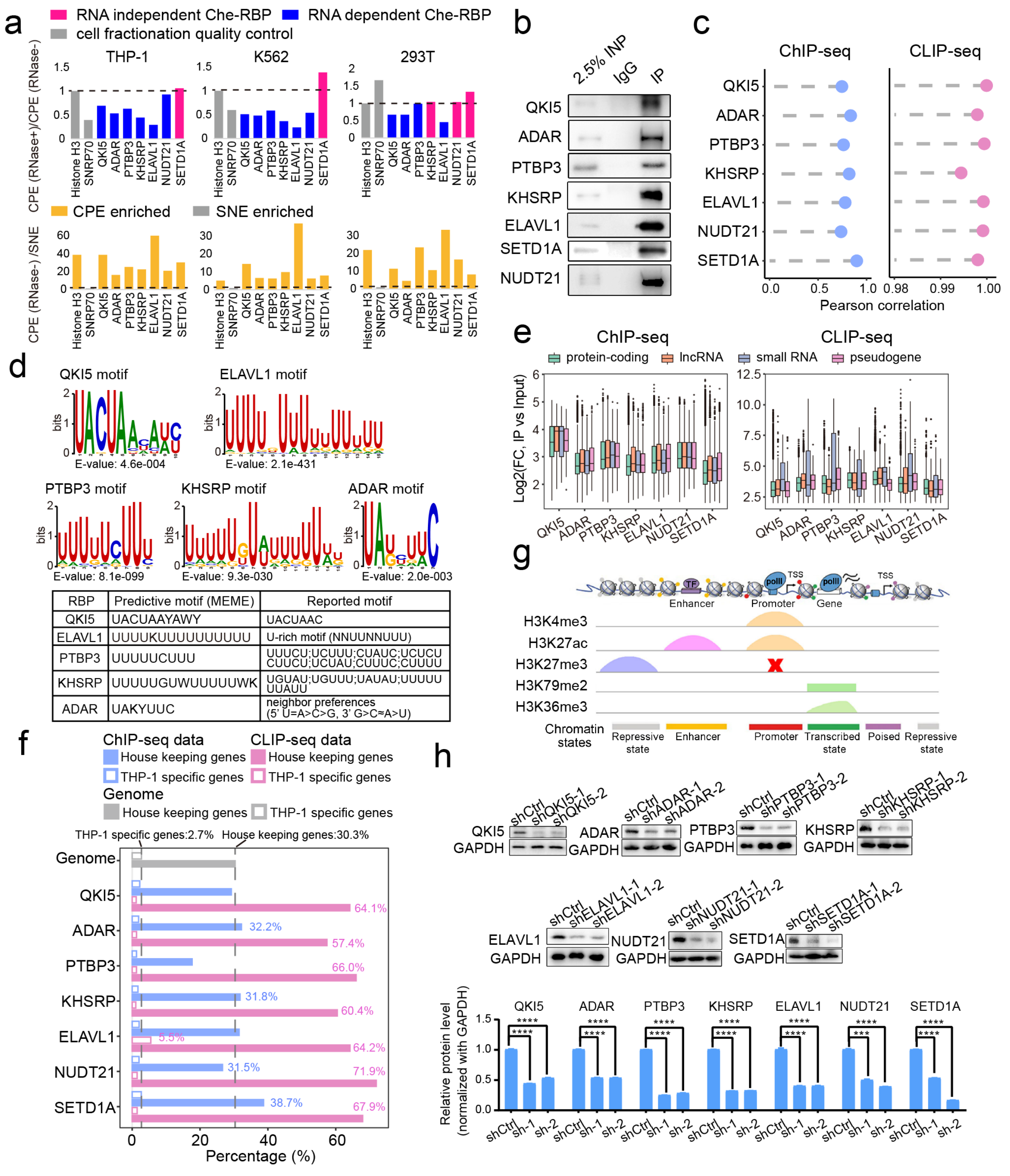

Figure S2. Large-scale sequencing of Che-RBPs. a Comparison of protein level quantified by quantitative analysis of immuno-blot results in Fig. 2b. RNA-dependency of Che-RBPs' deposition on chromatin was calculated as the ratio of $\frac{\text{Protain CPE (RNase+)/Protein CPE (RNase−)}}{\text{Histone H3 CPE (RNase+)/Histone H3 CPE(RNase−)}}$. RNA-independent Che-RBPs are indicated by pink bars with $\frac{\text{Protain CPE (RNase+)/Protein CPE (RNase−)}}{\text{Histone H3 CPE (RNase+)/Histone H3 CPE(RNase−)}}$>1. RNA-dependent Che-RBPs are indicated by blue

bars with $\frac{\text{Protain CPE (RNase+)/Protein CPE (RNase}-)}{\text{Histone H3 CPE (RNase+)/Histone H3 CPE(RNase}-)}$<1 (upper panel). Sub-nuclear localization of Che-RBPs was determined by $\frac{\text{Protein CPE (RNase}-)}{\text{Protein SNE}}$ > 1 (lower panel). **b** Immuno-blots of hChe-RBPs immunoprecipitated using indicated antibodies. IgG was used as negative control of immunoprecipitation assay. **c** Repeatability test of ChIP-seq (left panel) and CLIP-seq (right panel) assays. Pearson correlation coefficient was used to evaluate the repeatability of two biology replicates in ChIP-seq and CLIP-seq datasets. **d** CLIP motif prediction of indicated hChe-RBPs by MEME. The table below shows the reported CLIP motifs of the hChe-RBPs compared with motifs generated from our CLIP datasets. **e** hChe-RBPs' enrichment on different gene types calculated by log2 FC (IP vs. Input) of ChIP-seq (left panel) and CLIP-seq (right panel) peaks. (FC: fold change). **f** hChe-RBPs' occupation frequencies on DNA or RNA transcripts of house-keeping (HK) genes and cell-type specific (SP) genes in THP-1 cells. Blue bars represent ChIP-seq results and pink bars indicate CLIP-seq results. **g** Diagram of the distribution of the indicated histone modifications in different chromatin states/regions. **h** Immuno-blot of indicated hChe-RBP expression level upon shCtrl or specific shRNA treatment. Quantitative analysis of each Che-RBP's level from immune blots is shown below. Error bars indicate standard deviations around the mean of three experimental replicates. Asterisks indicate a significant difference between the specified samples (****P*-value<0.001, *****P*-value<0.0001, t test).

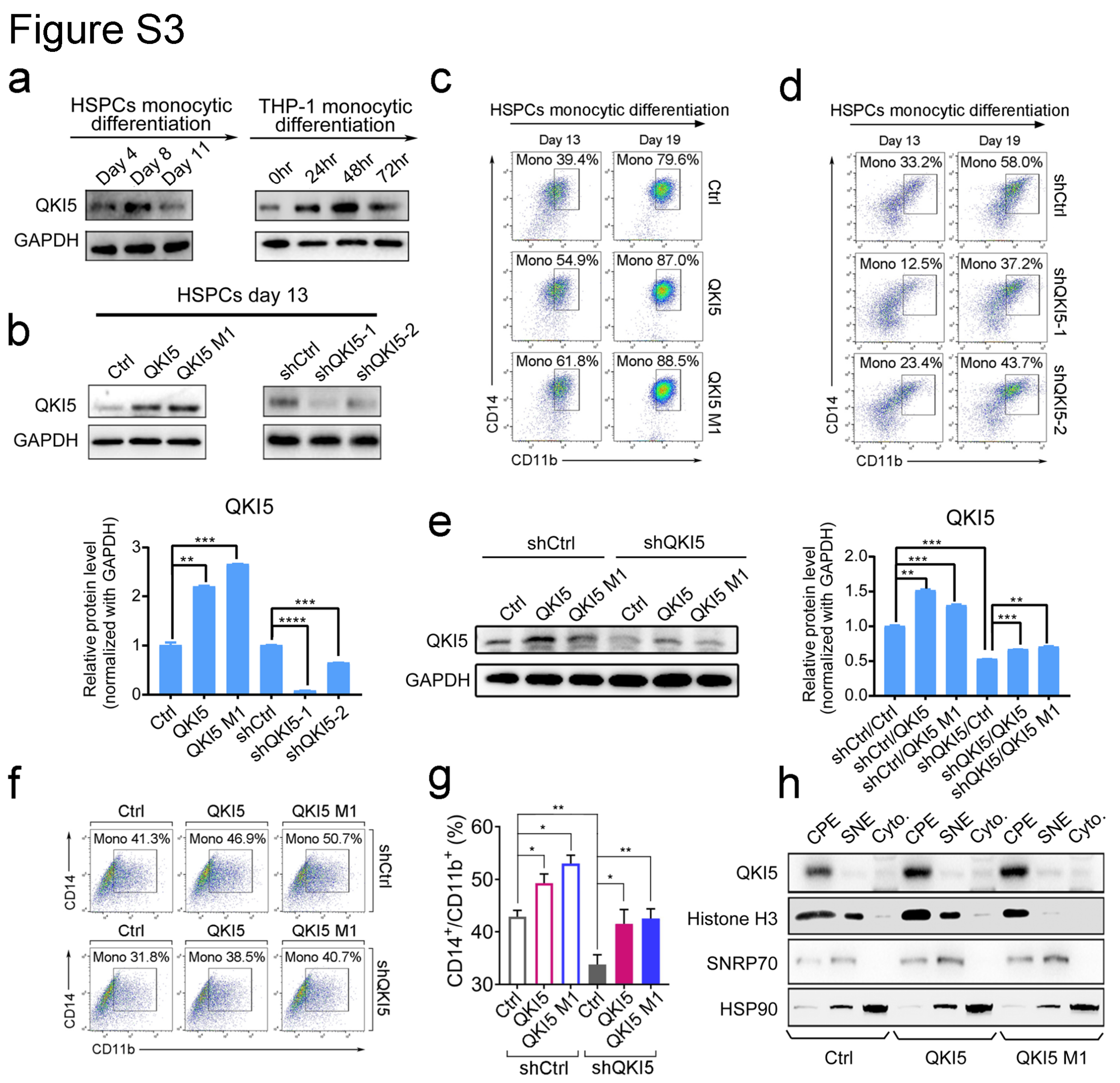

Figure S3. QKI5 promotes monocytic differentiation in both HSPCs and THP-1 cells. a Detection of QKI5 protein during monocytic differentiation in HSPCs (left panel) and THP-1 cells (right panel). **b** Immuno-blot detection of QKI5 in Ctrl- or QKI5/QKI5 M1-overexpressing and knockdown HSPCs with quantitative analysis below. Error bars indicate standard deviations around the mean of three experimental replicates. Asterisks indicate a significant difference between the specified samples (***P*-value<0.01, ****P*-value<0.001, *****P*-value<0.0001, t test). **c** Percentage of $CD14^+/CD11b^+$ cells among HSPCs within Ctrl- or QKI5/QKI5 M1-overexpressing population during monocytic differentiation, detected by flow cytometry. **d** Percentage of $CD14^+/CD11b^+$ cells among HSPCs within shCtrl- or shQKI5-treated population during monocytic differentiation, detected by flow cytometry. **e-g** QKI5 rescue assay in THP-1 cells during monocytic differentiation. **e** Immuno-blot of QKI5 levels in the rescue assay with quantitative analysis shown on the right. Error bars indicate standard deviations around the mean of three experimental replicates. Asterisks indicate a significant difference between the specified samples (***P*-value<0.01, ****P*-value<0.001, t test). **f** Percentage of $CD14^+/CD11b^+$ cells

among THP-1 cells within shCtrl- or shQKI5-treated population, followed by Ctrl or QKI5/QKI5 M1 overexpression and subsequent PMA induction for 48hr, detected by flow cytometry. **g** Average percentage of $CD14^{+}/CD11b^{+}$ in cells from (**f**), error bars indicate standard deviations around the mean of three biological replicates. Asterisks indicate significant differences between the specified samples (**P*-value < 0.05, ***P*-value<0.01, t test). **h** Validation of the sub-cellular fractionation detection of QKI5 in Ctrl- and QKI5/QKI5 M1-overexpressing THP-1 cells.

Figure S4

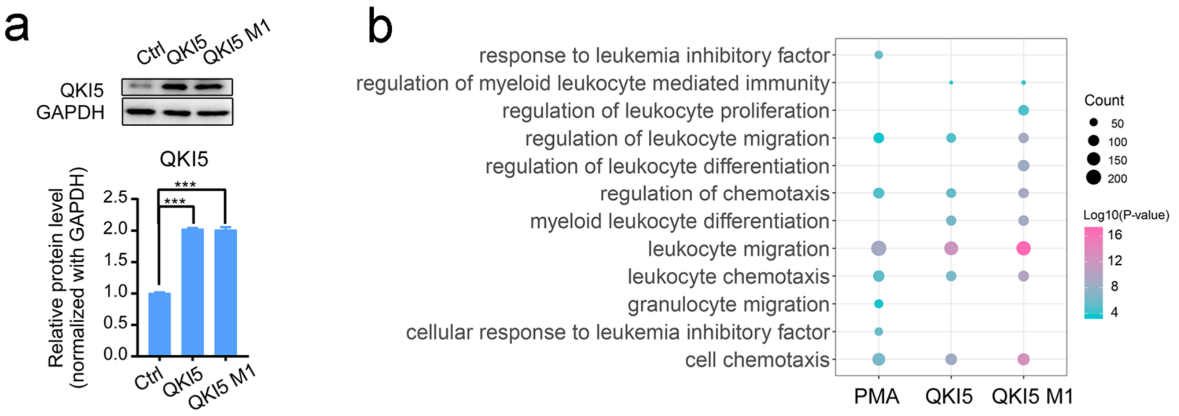

Figure S4. QKI5 influences monocytic differentiation-related gene expression. a Immuno-blot of QKI5 levels in Ctrl or QKI5/QKI5 M1 over-expressing THP-1 cells used for RNA-seq. Quantitative analysis is shown below. Error bars indicate standard deviations around the mean of three experimental replicates. Asterisks indicate a significant difference between the specified samples (****P*-value<0.001, t test). **b** GO functional enrichment analysis of QKI5, QKI5 M1-activated genes and PMA-activated genes. The color and size of the bubble indicate the log10 (*P*-value) and gene counts of the GO term, respectively.

Figure S5

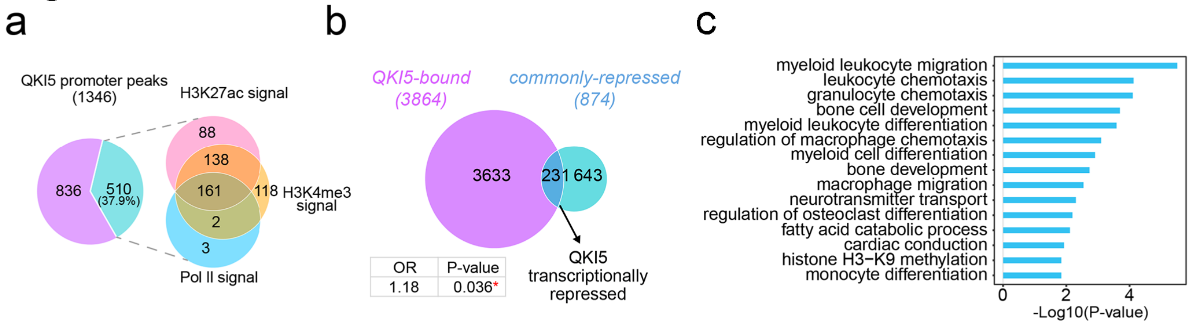

Figure S5. QKI5 preferentially binds to activated gene promoters. a Pie chart showing the number of QKI5 ChIP-seq peaks on promoter regions with/without H3K4me3/H3K27ac/Pol II ChIP-seq peaks. And the intersection of ChIP-seq peaks of H3K27ac, H3K4me3 and Pol II on QKI5 promoter peaks region is listed on right Venn diagram. **b** Venn diagram showing the intersection of QKI5-bound genes and QKI5/QKI5 M1/PMA commonly-repressed genes, *P*-value was calculated by double-tailed Fisher' exact test (**P*-value < 0.05). **c** GO functional enrichment analysis of the intersection of genes generated by QKI5 ChIP-targets genes and QKI5 commonly-activated genes.

Figure S6

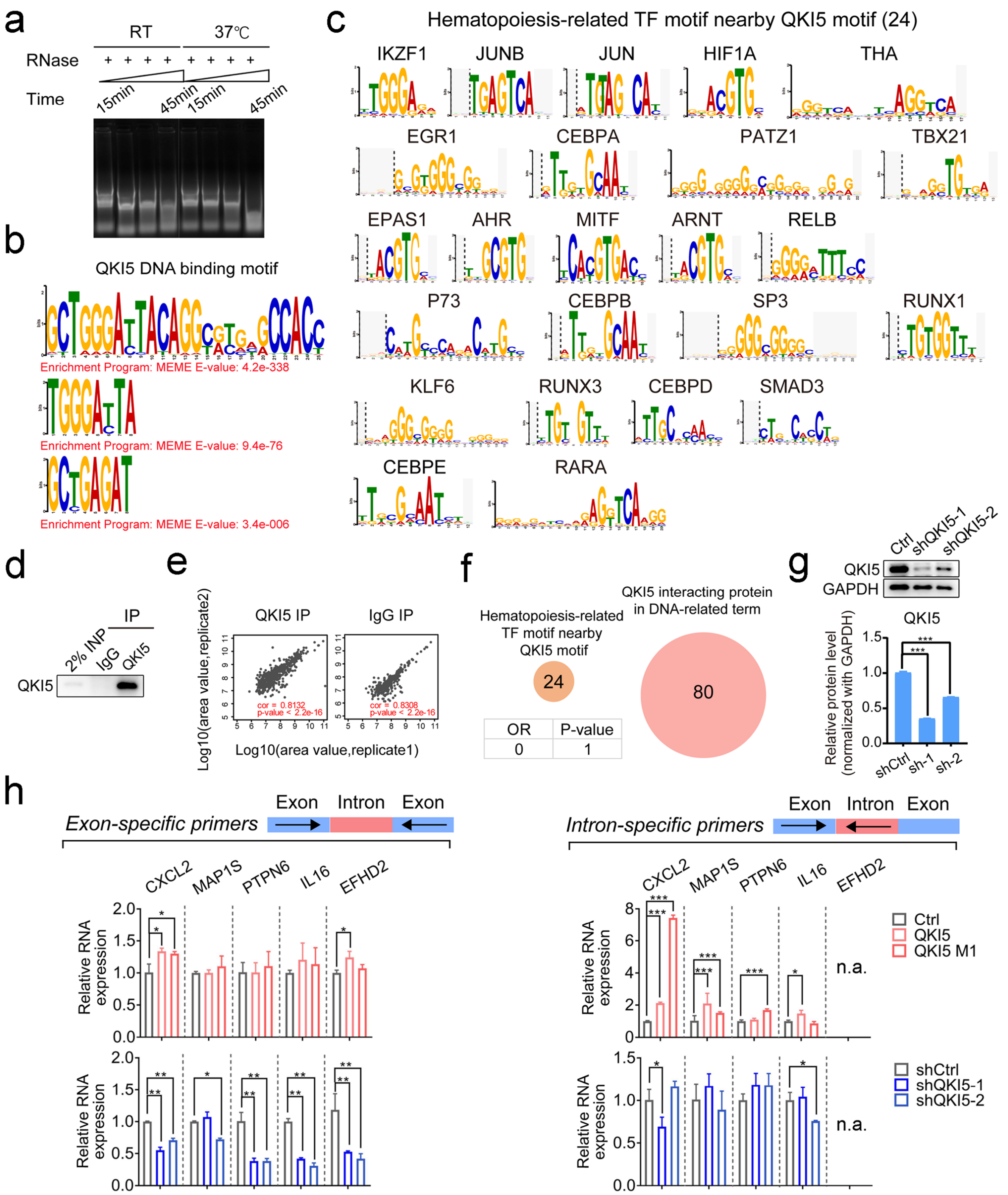

Figure S6. QKI5 associates directly with chromatin. a Efficiency test for RNase treatment in ChIP-qPCR assay. THP-1 cells were permeabilized and subjected to RNase A treatment for up to 45 minutes at room temperature or 37°C. The agarose gel picture shows the varying degrees of digestion. **b** QKI5 DNA binding motif predicted by the MEME online suite. **c** 24 hematopoietic-related TF motifs nearby the QKI5 motif predicted by SpaMo. **d** Immuno-blot of QKI5 immunoprecipitated by

QKI5 or IgG antibody as used for MS analysis. **e** Repeatability test of QKI5 co-immunoprecipitation (QKI co-IP) MS results. Pearson correlation coefficient was used to evaluate the repeatability of two biology replicates in QKI5 co-IP and IgG co-IP data. **f** Venn diagram showing the overlap between TFs in Figure 6c and proteins interacting with QKI5 in DNA-related terms identified by co-IP MS analysis. **g** Immuno-blot of QKI5 level in shCtrl-, shQKI5-1/ shQKI5-2-treated THP-1 cells used in the nuclear run-on assay. Quantitative analysis is shown below. Error bars indicate standard deviations around the mean of three experimental replicates. Asterisks indicate a significant difference between the specified samples (****P*-value<0.001, t test). **h** RT-qPCR validation of expression of selected target genes using exon-specific (left panel) and intron-specific (right panel) primers, respectively. qPCR was performed in Ctrl or QKI5/QKI5 M1-overexpressing (upper panel) and shCtrl- or shQKI5-treated (lower panel) THP-1 cells (without PMA induction). Error bars indicate standard deviations around the mean of three biological replicates. Asterisks indicated significant differences between the specified samples (**P*-value < 0.05, ***P*-value<0.01, ****P*-value<0.001, n.a. represents not available, t test).

Figure S7

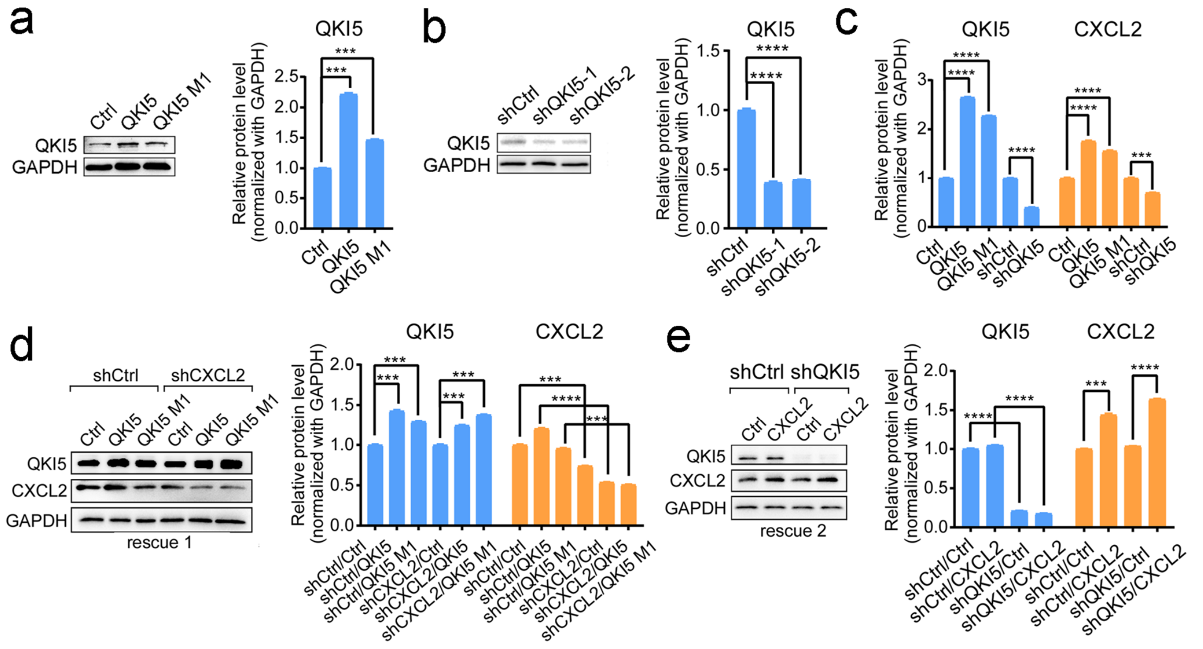

Figure S7. The level of QKI5 and CXCL2 in rescue assays. a Immuno-blot of QKI5 protein levels in Ctrl and QKI5/QKI5 M1 over-expressing 293T cells used in the dual luciferase assay. Quantitative analysis is shown on the right. **b** Immuno-blot of QKI5 protein levels in shCtrl-, shQKI5-1/shQKI5-2-treated 293T cells used in the dual luciferase assay. Quantitative analysis is shown on the right. **c** Quantitative analysis for corresponding immuno-blot in Fig. 7d. **d** Immuno-blot of CXCL2 and QKI5 protein expression in rescue 1. Quantitative analysis is shown on the right. **e** Immuno-blot of CXCL2 and QKI5 protein expression in rescue 2. Quantitative analysis is shown on the right. Error bars indicate standard deviations around the mean of three experimental replicates. Asterisks indicate a significant difference between the specified samples (****P*-value<0.001, *****P*-value<0.0001, t test).

Raw blots images in Figures

Fig. 1h

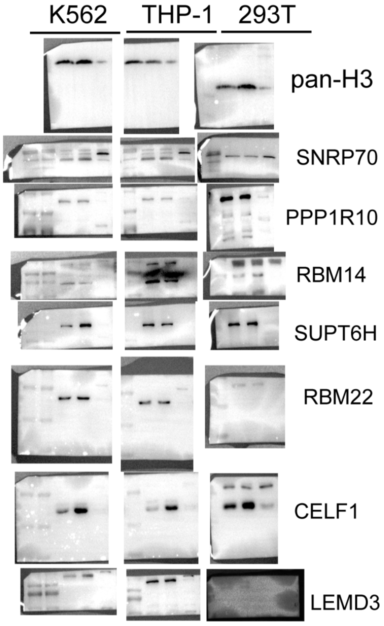

Fig. 2b

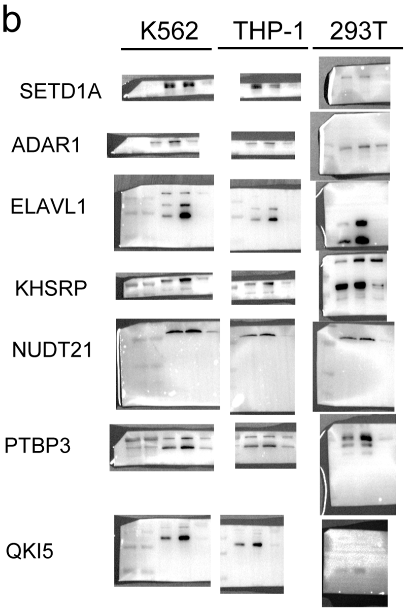

Fig. 7d

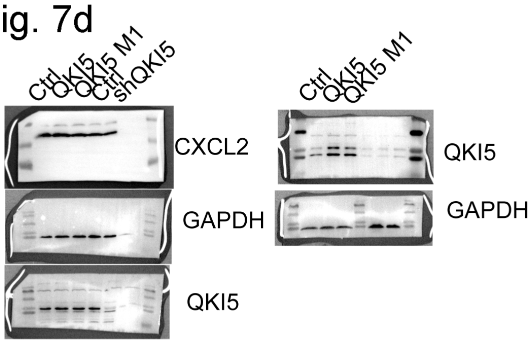

Raw blots images in Supplementary Figures

Fig. S1a

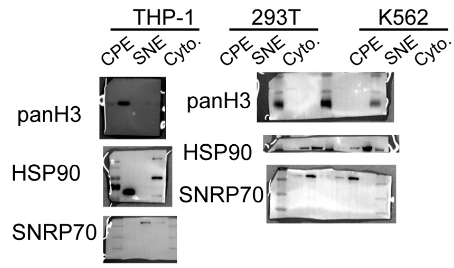

Fig. S2b

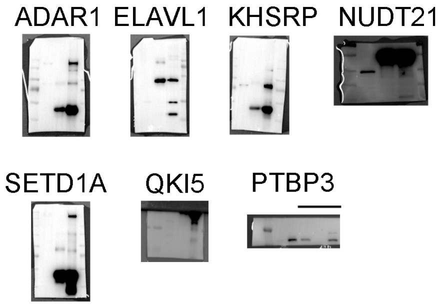

Fig. S2h

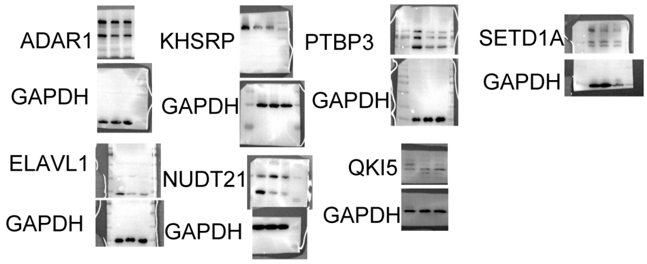

Fig. S3a

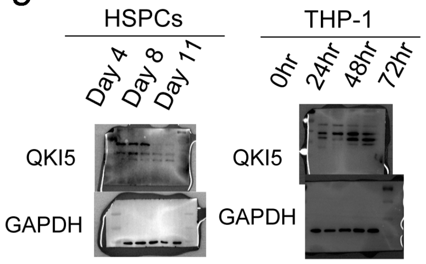

Fig. S3b

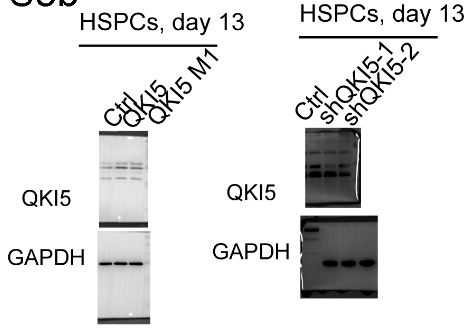

Fig. S3e

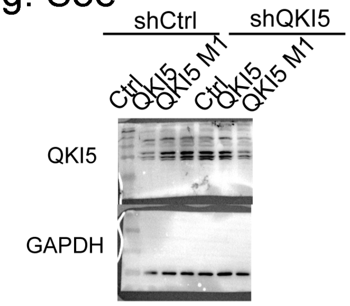

Fig. S3h

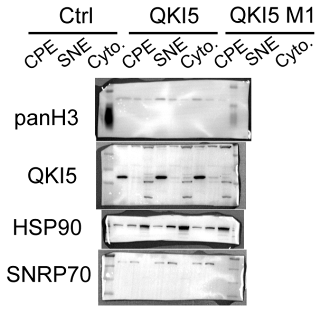

Fig. S4a

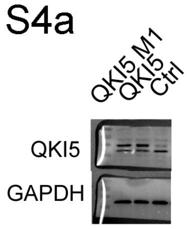

Fig. S6d

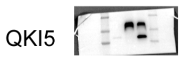

Fig. S6g

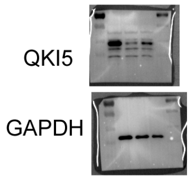

Fig. S7d

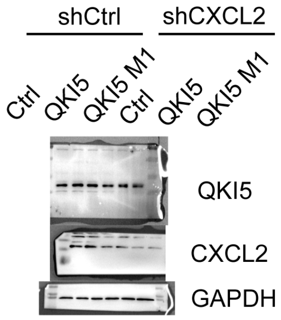

Fig. S7e

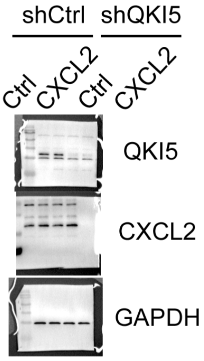

Fig. S7a

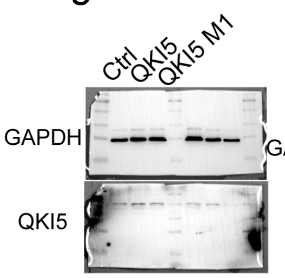

Fig. S7b

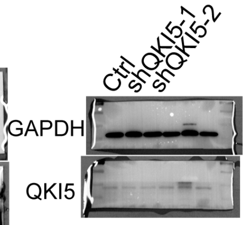

Supplement: Supplementary file 1 — Additional file 1: Figure S1-S7 and raw blot images in Figures and Supplementary Figures [file 13059_2021_2508_MOESM1_ESM.pdf]
